# The Fox and the Rabbits—Environmental Variables and Population Genetics (1) Replication Problems in Association Studies and the Untapped Power of GWAS (2) Vitamin A Deficiency, Herpes Simplex Reactivation and Other Causes of Alzheimer's Disease

**DOI:** 10.5402/2011/394678

**Published:** 2011-07-12

**Authors:** C. J. Carter

**Affiliations:** PolygenicPathways, Flat 4, 20 Upper Maze Hill, St Leonards-on-Sea, East Sussex, TN38 0LG, UK

## Abstract

Classical population genetics shows that varying permutations of genes and risk factors permit or disallow the effects of causative agents, depending on circumstance. For example, genes and environment determine whether a fox kills black or white rabbits on snow or black ash covered islands. Risk promoting effects are different on each island, but obscured by meta-analysis or GWAS data from both islands, unless partitioned by different contributory factors. In Alzheimer's disease, the foxes appear to be herpes, borrelia or chlamydial infection, hypercholesterolemia, hyperhomocysteinaemia, diabetes, cerebral hypoperfusion, oestrogen depletion, or vitamin A deficiency, all of which promote beta-amyloid deposition in animal models—without the aid of gene variants. All relate to risk factors and subsets of susceptibility genes, which condition their effects. All are less prevalent in convents, where nuns appear less susceptible to the ravages of ageing. Antagonism of the antimicrobial properties of beta-amyloid by Abeta autoantibodies in the ageing population, likely generated by antibodies raised to beta-amyloid/pathogen protein homologues, may play a role in this scenario. These agents are treatable by diet and drugs, vitamin supplementation, pathogen detection and elimination, and autoantibody removal, although again, the beneficial effects of individual treatments may be tempered by genes and environment.

## 1. Introduction

If there is one factor common to complex polygenic diseases it is the heterogeneity in both gene and risk factor association studies. 

 Although these have discovered key genes and risk factors, the results for most are invariably confounded by conflicting data [1]. In the genetic arena, the clear familial component of many diseases has driven the search for major genes using genome-wide association studies (GWAS) with large numbers of patients pooled from different regions [2]. Such studies have been able to discover rare variants that play a major role in a small percentage of patients, for example VIPR2 in schizophrenia [3]. However, in complex diseases, these have failed to find major genes relevant to all patients [4], instead unearthing yet more genes of small effect, whose risk promoting effects are yet again contested, as is the case with CR1 and PICALM, which have not been confirmed as risk factors for Alzheimer's disease in Chinese patients [5] despite extensive evidence in Caucasian studies [6]. GWAS studies have, however, been more successful in uncovering larger numbers of genes of greater effect for simpler traits such as lipid levels [7].

Viruses and other pathogens have been implicated as risk factors in many diseases, although again, conflicting evidence leads to scepticism in many areas. For example, the involvement of the Epstein-Barr virus in multiple sclerosis is hotly contested [8–10]. 

Gene-gene and gene-environment interactions may play an important role in such inconsistency. For example, the risk promoting effects of genes can be better explained when using pathway analysis or combining the effects of genes with common function, rather than by studying single genes in isolation [11, 12]. Genes and risk factors can also act together, and in certain cases genes can be linked to environmental variables. For example, many of the genes implicated in schizophrenia or Alzheimer's disease are involved in the life cycles of the pathogens involved in the diseases [13, 14]. Environment-environment interactions are also apparent. For example, the effects of vitamin E on lifespan, or on resistance to various infections can be null, deleterious, or protective, depending on confounding factors such as age, exercise, smoking, and vitamin C consumption [15–17]. 

Complex diseases are also composed of many endophenotypes or underlying pathologies, and different genes or risk factors may contribute to any of these. Many different processes contribute to cell death in Alzheimer's disease, for example, beta amyloid, glutamate, calcium, or free radical mediated toxicity [18, 19]. The efficiency of each of these subprocesses is controlled by genes, many of which have been implicated in association studies (see Table 1).

In genetic association studies, the drive has been to increase statistical power by increasing the numbers of subjects enrolled. This has resulted in the discovery of important genes and rare genetic variants, but has not delivered genes that confer a high degree of risk in the majority of patients. However, as illustrated below, more could perhaps be gained by a reanalysis of existing data in relation to other genetic and risk factor variables that could result in elucidation of the causes rather than the risks.

## 2. Methods

Alzheimer's disease susceptibility genes and risk factors are stocked in an online database at http://www.polygenicpathways.co.uk/alzenvrisk.htm. KEGG pathway analysis of over 400 associated genes was performed [20], and the results of the exercise were posted at http://www.polygenicpathways.co.uk/alzkegg.htm. In these figures, yellow genes have been implicated in Alzheimer's disease and red genes are also implicated in the herpes simplex life cycle. Other gene-risk factor relationships were identified by literature survey. B cell and T cell epitopes within the beta-amyloid peptide were identified using the immune epitope database server http://tools.immuneepitope .org/main/index.html which predicts the antigenicity of peptide sequences, based on their charge and hydrophobicity properties [21]. Sequence comparisons of the beta-amyloid peptide versus selected bacterial, fungal, or viral proteomes was performed using the NCBI blast server [22, 23].

## 3. Results and Discussion

### 3.1. Kegg Pathway Analysis of Alzheimer's Disease Susceptibility Genes

The overall results of this analysis are shown in Table 1. The pathways include many that are relevant to the known pathologies and risk factors of Alzheimer's disease, including the Alzheimer's disease pathway itself, primarily related to beta-amyloid and *tau* processing, but also to glutamate-related pathways (long-term potentiation and depression), apoptosis, insulin and diabetes pathways, neurotrophin signalling, oxidative stress (glutathione/oxidative phosphorylation), cerebral hypometabolism (oxidative phosphorylation, glycolysis and the Krebs cycle), arginine and proline metabolism (including nitric oxide), and folate, methionine and homocysteine metabolism, and steroid hormone synthesis (together with androgen and oestrogen receptors AR, ESR1, and ESR2). PPAR signalling regulates many lipoprotein-related genes and cholesterol/lipid pathways are dispersed in terpenoid backbone biosynthesis (FDPS, HMGCR, HMGCS2), steroid hormone biosynthesis (HSD11B1), steroid biosynthesis (DHCR24, LIPA, SOAT1), glycerolipid metabolism (ALDH2, LIPC, LPL), and bile acid biosynthesis (CH25H, CYP46A1) pathways. Immune, complement, and cytokine-related pathways figure prominently, as do several pathogen defence pathways including the DNA sensing retinoic acid inducible gene (RIG-1) pathways that react to viral DNA/RNA by increasing the expression of interferons and other antiviral genes, and the Toll receptor and NOD pattern recognition pathway that control immune and cytokine networks [24–26]. Glutathione pathways were also present. Glutathione has potent viricidal and bactericidal properties and is often depleted by infections [27–30]. A number of pathogen entry pathways are also concerned, and although *C. Pneumoniae* or *C. Neoformans* pathways are not specifically represented, many of these pathways can be considered as generic pathways relevant to many bacteria and other pathogens. The *H. Pylori* pathway contains only two genes, IL8 and CSK, but others can be added to this list including all members of the PI 3-kinase/AKT signalling network which is activated by the *H. pylori* protein CagA [31] or Toll receptor pathways that are activated by *H. Pylori* heat shock protein, HSP60 [32]. Similarly, there is no specific HSV-1 viral entry pathway in the KEGG database, but the virus uses actin pathways, endocytosis, protein processing, and DNA repair pathways during its life cycle, which are heavily represented [33].

### 3.2. Vitamin-A-Related Genes

These were identified by literature survey and the most directly relevant are shown in Table 2. Of particular interest is a close relationship between cholesterol/lipoprotein-related genes and vitamin A. Both retinols and cholesterol are transported by lipoproteins, and the clusterin receptor, LRP2/megalin, is a key retinol entry point. APOE4 is the isoform least able to bind to retinyl palmitate. ABCA1 is also involved in cholesterol and retinol transport. Several genes (ALDH2, CYP46A1, GSTM1, GSTP1, LIPA, LPL, and LRAT) are involved in Vitamin A metabolism, and 24-s hydroxycholesterol, the product of CYP46A1, is a ligand for retinoic acid receptors (RARA and RARG). Retinoid coreceptors and binding partners include RXRA, ESR1, KLF5 NPAS2, NR1H2, PARP1, PIN1, POU2F1, PPARA, PPARG, THRA, UBQLN1, and VDR. Retinoids modulate APP processing, via regulation of beta and gamma secretases while the RIG-1 pathway is crucial in viral defence. A large number of genes are also regulated by retinoids or retinoid receptors.

### 3.3. The Fox, the Rabbits, Gene, and Environmental Variables

This is an adaptation of Lees's classical population genetics example of the peppered moth, whose dark or pale colouring confers advantage or disadvantage depending upon the degree of industrial pollution that covers trees with soot. It has served to illustrate the concept of natural selection where, over time, dark genes become more common in polluted areas, an effect that could eventually lead to speciation [34].

On two islands one covered in snow and the other in black volcanic ash live an equal number of black and white rabbits and a family of foxes, who will find it easier to trap the black rabbits on the snowy island and the white rabbits on the island covered in black ash. Gene association studies would correctly identify the black and white genes as being protective or risk promoting depending upon the environment. The snow, the ash, or the fox, being equally present on each island, regardless of the toll of dead rabbits, could not be considered as risk factors. Genetic meta-analysis or pooled GWAS data would also rule out any genetic involvement, leaving no susceptibility genes, no risk factors, and no cause. However, a GWAS study, apportioning the genetic data in relation to ash, snow, and fox would be able to correctly surmise that the white gene is a risk factor on the ash-covered island, and the black gene a risk factor on the snowy island, as would have D. R. Lees. Again the fox is undetectable, being present in all compartments. 

On other similar islands, live further populations of black and white rabbits with no fox, an equal number of deaths due to old age, and no reason to investigate either genes or risk factors. However, it is only by including this island, again partitioning GWAS data in relation to all variables, that the genes, the risk factors, and the cause can be correctly allotted their respective roles.

In this example, the genes and environmental variable are risk or protective factors for the cause as well as for the deaths, depending on circumstance. The genes or risk factors are not killing the rabbits, but are allowing the cause to do so. Nonstratified association studies would thus seem to be ill-suited to find important genes, risk factors, or causes, and the pursuit of greater statistical power may well be futile, although such strategies can find rare variants that may cause disease in a minority of patients, which is evidently useful. However, for the majority of cases, much could perhaps be gained from a reappraisal of existing data and by partitioning GWAS data in relation to the many known risk factors in each disease. 

The situation is evidently more complex in polygenic diseases, where hundreds of interacting genes, many risk factors, and probably many causes are present. This is already appreciated, and several groups have analysed the statistical problems involved due to the mass of genes and risk factors [35–37]. However, it is likely that an appropriate selection of genes and risk factors could markedly affect the degree of risk. For example, the odds ratio for APOE4 was shown to be 1.67 in Alzheimer's disease patients without cerebral HSV-1 DNA and 16.8 in patients where viral DNA was detected [38]. The population genetics example, and the discussion below, suggests that certain susceptibility genes are restricted to risk factor subsets.

From the above, it would appear that a cause can be present in equal proportion in control and disease populations but should be able to produce the pathological features of the disease, and the disease incidence should be reduced where the causes are few. A cause can kill regardless of the genes (black or white) or risk factors (snow or volcanic ash) but its effects are tempered by a combination of the two (fox + snow + black gene or fox + ash + white gene = death and fox + snow + white gene or fox + ash + black gene, or no fox and any combination = life). The genes and risk factors are, however, both able to influence the cause. 

In relation to Alzheimer's disease, beta-amyloid deposition can be produced by herpes simplex [189–191] or chlamydia pneumoniae infection [192, 193], hypercholesterolemia (which also causes cholinergic neuronal loss and memory deficits in rats [194–197]), by or hyperhomocysteinaemia, an effect reversed by folate and vitamin-B12 [198], by NGF deprivation [199], by reduced cerebral perfusion (hypoxia, cerebral ischaemia, or carotid artery occlusion [200–203]), as well as by experimental diabetes and streptozotocin [204, 205], oestrogen depletion [206], or vitamin A deficiency, which also reduces choline acetyltransferase activity in the forebrain [207]—all without the aid of any variant genes, in animal models. While none of the animal models faithfully reproduce the entire symptomatology of Alzheimer's disease, in the clinical setting these risk factors coexist to varying degrees, rendering the situation rather more complex.

The risk factors in Alzheimer's disease include herpes simplex infection [208] acting in combination with APOE4 [209], *C. Pneumoniae* [210, 211] or *Helicobacter pylori* infection [212, 213], mild hypercholesterolaemia [214], but declining cholesterol levels from midlife to late life [215], atherosclerosis of the carotid arteries, leptomeningeal arteries, or the circle of Willis, and stroke, leading to cerebral hypoperfusion [216–219], hyperhomocysteinaemia [220], type 2 diabetes and modified insulin metabolism [221], age-related loss of sex steroid hormones in both women and men [222], but high total oestradiol levels [223, 224], and vitamin A deficiency [225]. These factors may be confounded by interrelationships, and in some cases by the fact that death due to other causes—for example, atherosclerosis-related myocardial infarction or stroke may lead to an apparent paucity of comorbid risk factors in Alzheimer's disease groups at later ages. 

The genes implicated in Alzheimer's disease are related to the herpes simplex life cycle [13, 226], bacterial and viral entry pathways, viral and pathogen defence (Table 1), and the immune network [227], cholesterol and lipoprotein pathways [11, 228], folate and homocysteine pathways [229], and insulin, or neurotrophin signalling pathways, steroid metabolism and receptors (Table 1 and 2), and vitamin A metabolism and function (Table 2).

The genes, risk factors and agents known to increase beta-amyloid deposition all concur, suggesting that Alzheimer's disease is multifactorial with many foxes, each with their respective genes and risk factors, any of which can lead to beta-amyloid deposition in multifarious ways. Each risk factor can act independently of any gene or other risk factor variant, in animal models—as with the fox. This in turn might suggest that it is not the risk promoting polymorphisms in the Alzheimer's disease patients that are crucial, as the risk factors can in any case promote beta-amyloid deposition, but the equivalent polymorphisms in the control group, that are providing protection; a subtle distinction that awaits characterisation of the functional effects of many different gene variants. The reasoning also suggests that beta-amyloid deposition is the consequence and not the cause of the many factors able to promote Alzheimer's disease. Anoxia, ischaemia, hypoglycaemia, hypercholesterolaemia, and vitamin A deficiency are all able to kill neurones, in some cases including cholinergic neurones, without the aid of beta amyloid.

### 3.4. Low Incidence of Alzheimer's Disease and Protective Factors

In relation to Alzheimer's disease, there is an island where longevity is increased, related to the nun study [231–234]. Nuns do not have children, (the number of pregnancies is a risk factor in Alzheimer's disease [235]), do not consume high concentrations of saturated fats (low cholesterol), and are unlikely to have sexually transmitted diseases or viral and other common pathogen diseases vectored by childhood infections (Herpes and *chlamydia*, *inter alia*). Their vitamin A levels and general health are sustained by a healthy diet, regular fish on Fridays, and exercise.

There are few strategies that have been shown to reduce the severity of Alzheimer's disease, once established. It has, however, been shown that *Helicobacter pylori* elimination increases the cognitive abilities and the lifespan of Alzheimer's disease patients [236]. In addition, two separate case reports have shown complete reversal of dementia in two patients diagnosed with Alzheimer's disease, by identification and eradication of the fungal pathogen *Cryptococcus Neoformans* [237, 238].The TNF antagonist, etanercept, has also been reported to produce a striking remedial effect on symptomatology, following perispinal application [239]. However, the use of TNF antagonists is also associated with an increased incidence of opportunistic bacterial, fungal, and viral infestations, including cytomegalovirus, and cryptococcal infections [240], perhaps a contraindication for their prolonged use.

A number of epidemiological studies have shown that the incidence of Alzheimer's disease can be reduced, although, once the disease is established, there is little evidence for any curative effects of any treatment other than the above. These protective factors are in most cases the obverse of the risk factors and include diets rich in fish or polyunsaturated fatty acids [241, 242], the Mediterranean diet [243] and the use of statins [244], which are counter to the effects of high cholesterol. A diet rich in fruit and vegetables is associated with reduced dementia incidence [245] and is able to sustain Vitamin A levels and reduce homocysteine levels in the elderly population [246]. High folate intake, which reduces homocysteine levels [247], and the use of nonsteroidal anti-inflammatories have also been reported to reduce risk [248]. Again these are related to the risk factors and to the genes, which may condition their success (cf. Vitamin E).

### 3.5. Relevance of These Factors to the Genes Identified in Genome-Wide Association Studies

Four major genes have been discovered prior to and from GWAS studies, APOE4, clusterin, complement receptor 1, and PICALM [6, 249]. The close relationships between these genes and herpes simplex infection have been the subject of a previous article [226]. APOE4 also favours the binding of *C. Pneumoniae* elementary bodies to host cells [250]. It is also a risk factor for hypercholesterolaemia, *per se *[251], and for carotid artery atherosclerosis in men with diabetes [252]. APOE4 is also the isoform least able to promote lipid efflux from neuronal cells [253], a factor that may enhance the cholesterol dependent cleavage of beta-amyloid by beta and gamma secretase [254]. It is also the least able isoform binding the vitamin A precursor retinyl palmitate [255] (see below). Complement receptor 1 is a pathogen receptor for both herpes simplex [256], and *C. Neoformans* [257] and also for the atherogenic pathogen, *P. Gingivalis* [258], a key cause of periodontitis/gum disease, which has also been implicated as a risk factor in dementia [259]. Both *Helicobacter pylori* and *C. Pneumoniae* [260] use the mannose-6-phosphate IGF2 receptor (*inter alia*) for entry. This binds to clusterin and its endocytosis is controlled by PICALM [226]. PICALM (phosphatidylinositol binding clathrin assembly protein), as its name implies, is involved in clathrin-mediated endocytosis [261], a process used by *C. Pneumoniae* to gain cellular entry [262], for the internalisation of herpes simplex glycoprotein D [263] or the cytomegalovirus chemokine receptor [264] and for the uptake of outer membrane vesicles from certain strains of *H. pylori*, into gastric epithelial cells [265]. Clathrin also associates with HHV-6 virions [266]. Clusterin is an inhibitor of the membrane attack complex that is inserted into microbial membranes causing death by lysis [267]. It is also a ligand for the retinol/lipoprotein receptor LRP2 [268], and the gene contains a retinoid response element (Table 2). Thus it would seem that the key role of these genes may be related to their ability to target multiple aspects of diverse risk factor networks.

### 3.6. Relationships between Risk Factors and a Key Role for Herpes Simplex Activation (Figure 1)

Hypercholesterolaemia can evidently be related to other dietary risk factors such as saturated fat consumption, and to atherosclerosis. Docosahexaenoic acid increases total plasma cholesterol levels in hymans, but only in APOE4 carriers, an effect that may negate the cardioprotective effects of fish oil supplementation [269]. *Helicobacter pylori* infection also causes malabsorption of vitamin B12 and folate, leading to increased homocysteine levels, that can be restored by *H. pylori* eradication [270]. Homocysteine metabolism is also related to glutathione synthesis via the transsulfuration pathway (homocysteine→cysteine→glutathione). Increased levels of homocysteine and reduced levels of glutathione in Alzheimer's disease suggest impairments in the transsulfuration pathway [271]. Glutathione is a potent antiviral and bactericidal agent with effects targeted at herpes simplex and C. Pneumoniae*, inter alia* both of which also diminish glutathione levels in infected cells [27, 272, 273]. H. pylori expresses an enzyme, gamma-glutamyltranspeptidase that enables it to metabolise the host's extracellular glutamine and glutathione which are hydrolysed to glutamate, which is fed into the H. pylori Krebs cycle, resulting in diminished glutathione levels that can be restored by H. pylori elimination [27, 273]. Glutathione levels are reduced in Alzheimer's disease and many others [274]. 

In both coronary artery disease and carotid artery atherosclerosis, high plasma levels of homocysteine are positively correlated with *C. Pneumoniae* seropositivity suggesting a role for the bacterium in promoting high homocysteine levels. [275, 276]. Indeed carotid artery atherosclerosis is correlated with antibodies to *C. Pneumoniae*, and to a lesser extent with antibodies to *H. pylori*, but in this case, only in individuals with low social status [277]. 

The growth of C. Neoformans is attenuated by diethylstilbestrol and oestradiol but not by progesterone or testosterone [278]. Helicobacter pylori adsorbs a number of steroids including pregnenolone and two androgens (dehydroepiandrosterone and epiandrosterone and 3-hydroxylated oestrogens (oestrone and oestradiol). These are glucosylated and the glucosyl-steroid hormone derivatives used as membrane lipid components [279]. Oestradiol, androstenedione, and progesterone are all able to inhibit the growth of H. pylori [280]. 

These complex interactions, of which there are likely many more, suggest that in addition to epistasis and gene-environment interactions, environment-environment interactions have to be factored in to an already complex equation (cf. vitamin E). 

Factors known to reactivate herpes simplex include heat [281], 17-beta oestradiol [282] and the inflammatory cytokine interleukin 6 [283] where a role for corticosterone has been proposed [284]. NGF deprivation [285] also reactivates the virus and NGF promotes viral latency via the TrkA receptor [286] (cf. neurotrophin signalling). Vitamin A supplementation in rats increases the cerebral levels of both NGF and BDNF [129] while oestrogen deficiency lowers cerebral NGF levels, an effect reversed by 17-beta oestradiol [287]. Transient cerebral ischaemia lowers NGF levels [288]. Hypoxia is also able to increase the replication of herpes simplex [289].

Fevers induced by diverse infections might thus be expected to reactivate herpes simplex, as well as cerebral hypoperfusion. IL6 plasma and CSF levels have been reported to be increased in Alzheimer's disease and the secretion of IL6 from monocytes is increased [290–292]. IL6 plasma levels are raised by infection with *C. Pneumoniae* [293] or *Helicobacter pylori* [294], and IL6 production in monocytes is stimulated by *C. Neoformans* [295]. Cortisol levels are also increased in the ageing population and in Alzheimer's disease [296, 297]. High levels of total oestradiol have been reported as a risk factor for Alzheimer's disease in both women and men [223, 224]. 

As so many other risk factors seem able to reactivate the virus, this may be a key precipitant for the final curtain. Herpes simplex viral DNA is present in Alzheimer's disease plaques [298], and the plaques and tangles in Alzheimer's disease contain a very high proportion of herpes simplex interacting proteins, as well as immune-related components. The presence of the complement membrane attack complex in neurones suggests that the neuronal destruction in Alzheimer's disease might well be related to the consequences of battle between the virus and the immune network that has eliminated the virus at a terrible cost of collateral neuronal damage [33]. IgM+ antibodies, which preferentially index HSV-1 reactivation, have been shown to be able to predict the future risk of developing Alzheimer's disease [208], and the ability of other risk factors, particularly other infections, to reactivate the virus suggests a complex interplay of genes and risk factors that funnel towards viral reactivation and plaque and tangle formation.

### 3.7. The Importance of Vitamin A

Low vitamin A levels are a problem in the ageing population, and even in successfully ageing persons can be observed in 50% of the population over the age of 80–85 [299] (cf. the fox). Low vitamin A levels are also a risk factor for Alzheimer's disease [225]. Vitamin A plays an important role in maintaining the immune system [300], many genes of which are implicated in Alzheimer's disease. APP is involved in the vitamin A arena, as a gamma57 gamma secretase cleavage product suppresses retinoid signalling [75]. 

The vitamin A derivative, retinoic acid, inhibits herpes simplex replication [301, 302] as well as chlamydial infection and growth [303]. Vitamin A also stunts the growth of *Helicobacter pylori* [304]. The effects of vitamin A on *C. Neoformans* do not appear to have been examined. However, glucuronoxylomannan, the polysaccharide component of the capsular material of cryptococcus neoformans, exhibits potent immunosuppressive properties. This compound downregulates TNF-alpha and IL-1beta, and upregulates the inhibitory cytokine IL-10, but also inhibits retinoic receptor (RORG) synthesis [305]. Retinoic acid is also able to lower plasma homocysteine levels via the induction of hepatic glycine N-methyltransferase. Homocysteine in contrast inhibits retinoic acid synthesis [306, 307]. 

Vitamin A levels are in fact higher in hypercholesterolemia patients [308]. This may perhaps be due to the fact that retinyl palmitate, the vitamin A precursor, like cholesterol, is also transported by lipoproteins in the blood, mainly in the VLDL fraction (which primarily consist of APOC2/APOE) and the LDL fraction (which primarily consists of APOB) [41]. Retinyl palmitate concentrations in the blood are affected by APOE polymorphisms and radiolabelled retinyl palmitate binding in total plasma and nonchylomicron fractions is least in APOE4+/+ carriers [255]. The aortic concentrations of triglycerides, total cholesterol, free and esterified cholesterol, and phospholipids are increased in vitamin A deficient rats, an effect reversed by vitamin A supplementation [144]

A large number of Alzheimer's disease genes are related to vitamin A (Table 2), and an even larger number responsive to retinoic acid via the action of RAR or RXR transcriptional control.

### 3.8. Autoantibodies to the Antimicrobial Peptide Beta Amyloid: Likely Derivation from Antibodies to Pathogens

Beta-amyloid is a potent antimicrobial peptide. Although not tested against *C. Neoformans*, *H. pylori*, or *C. Pneumoniae*, it was found to have broad spectrum activity against a variety of yeasts and bacteria, effects that were attenuated by anti-A*β* antibodies. [309]. Lactotransferrin is also an antimicrobial peptide that colocalises to plaques and tangle in the Alzheimer's disease brain [310] and other antimicrobial peptides include the susceptibility gene products cystatin C, defensin DEFB122, myeloperoxidase, and transferrin [88]. beta-amyloid, like acyclovir, also attenuates the stimulatory effects of HSV-1 on miRNA-146a levels in neuronal cells [311]. The antimicrobial and antiviral properties of beta amyloid are, however, likely to be abrogated by the presence of beta-amyloid antibodies in the sera of the ageing population [312] and in Alzheimer's disease [313]. 

As shown in Table 3, a number of pathogens implicated in Alzheimer's disease or its attendant risk factors, express proteins with a high degree of homology to beta-amyloid. These include proteins from HSV-1, HHV-6, the cytomegalovirus, *C. Neoformans*, *H. pylori*, *C. Pneumoniae*, *B. Burgdorferi and, P. Gingivalis.* Antibodies to *B. Burgdorferi* [314] and *C. Pneumoniae* [210] and Herpes simplex and HHV-6 viral DNA [298, 315] have all been reported in or around Alzheimer's disease plaques, and antibodies to *H. pylori* recovered from Alzheimer's disease serum and cerebrospinal fluid [316]. A recent study showed that *P. Gingivalis* antibodies, cross-reactive with human HSP60, were observed in 100% of a sample of 20 atherosclerosis patients [317]. The immune system is not designed to raise antibodies to a self-protein, and this high degree of homology suggests that the autoantibodies to beta-amyloid are created by antibodies to homologous pathogens' proteins. 

Several other autoantibodies have been reported in Alzheimer's disease, including targets such as nerve growth factor [318], cholinergic neurones [319], the choroid plexus [320], and neurofibrillary tangles, *inter alia* [321]. The very extensive sharing of viral and bacterial protein sequences with the human proteome [230, 322–324] suggests that these too might be derived from cross-reactive microbial antigens. Again the ability to create autoantibodies is conditioned by genes, particularly HLA-antigens [325, 326]. Autoantibodies are often regarded as an epiphenomenon, but their ability to traverse the blood-brain barrier [327], and the recent recognition of their ability to enter cells [328] casts them in a new light as pathological immunopharmacological agents able to block the function of their target proteins: this has indeed been shown for Alzheimer's disease-derived ATP synthase autoantibodies which block ATP synthesis and cause apoptosis in neuroblastoma cells [329].

## 4. Summary

The classical population genetics example of the foxes and rabbits illustrates how genes and risk factors can differentially permit or disallow the effects of a causative agent, depending on a permutation of circumstance. Applying this model to Alzheimer's disease also suggests that many of the environmental risk factors in Alzheimer's disease are in fact causative agents, at least in terms of an ability to produce beta-amyloid deposition, *per se*, as shown in nontransgenic animal models. Their effects are also clearly related to other risk factors and genes. This would infer that the susceptibility genes in Alzheimer's disease permit the actions of these agents but, perhaps more importantly, that polymorphisms in the control population do not. Functional characterisation of these control variants may provide important clues to overall methods of protection. 

Many of these risk factors are avoidable or amenable to therapy. Diet is already known to be an important risk/protective factor with regard to the incidence of Alzheimer's disease. For example there is clear evidence that the omega-3 fatty acid docosahexaenoic acid (DHA), a component of fish and the Mediterranean diet, also protective factors [243, 330], is associated with a reduced risk of dementia [331], although a recent study with DHA in mild to moderate Alzheimer's disease, failed to show disease arrest or diminution [332]. However, in agreement with epidemiology, DHA significantly benefited two measures of cognition in mild to moderate non-ApoE4 carriers [333]. High vitamin A and low homocysteine levels are related to a high intake of fruit and vegetables in elderly patients [246]. Fruit and vegetable juice consumption is also associated with reduced Alzheimer's disease incidence [334].

Given the fact that the potential causes of Alzheimer's disease appear to be multifactorial, perhaps a multifactorial therapeutic effort is also needed. Such approaches might include a greater attention to diet, homocysteine and cholesterol levels, vitamin A supplementation where necessary, and the regular detection and elimination of herpes simplex, *B. Burgdorferi, C. Pneumoniae*, *H. pylori*, *C. Neoformans,* and other pathogens in the ageing population. The removal of beta-amyloid antibodies and of others prevalent in Alzheimer's disease might also be of benefit. These are simple preventive measures, requiring public health attention, whose *corporate* instigation might markedly reduce the incidence, and perhaps halt or reverse the progression, of Alzheimer's disease.

## Figures and Tables

**Figure 1:**
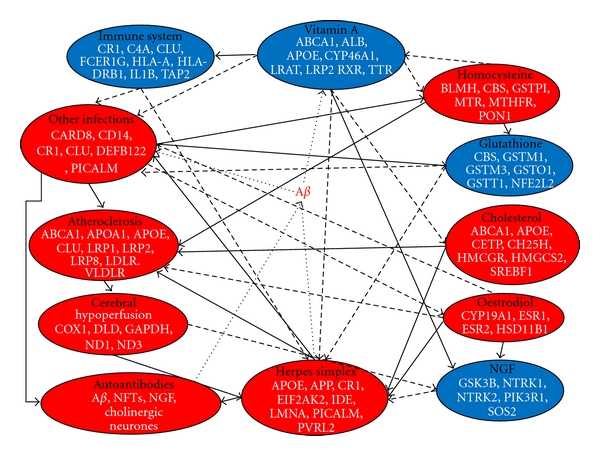
An environmental risk factor-gene interactome in Alzheimer's disease. Risk factors diminished in Alzheimer's disease (vitamin A deficiency, NGF levels, immune competence, and glutathione depletion) are shown in blue and those increased in red. Solid lines indicate a positive, and dashed or dotted lines a negative effect of risk factor X on risk factor Y. All risk factors feed into increased beta-amyloid deposition. A selection of susceptibility genes relevant to each process is shown (see Table 2 and http://www.polygenicpathways.co.uk/alzpolys.html.

**Table 1:** A summary of the KEGG pathway analysis of Alzheimer's disease susceptibility genes. The number of genes in each pathway is shown in brackets (see http://www.polygenicpathways.co.uk/alzkegg.htm for coloured figures).

Immune system and pathogen defence	Pathogen entry pathways	Structural and DNA repair (and HSV-1 pathways)
Cytokine-cytokine receptor interaction (13)	Chagas disease (17)	Regulation of actin cytoskeleton (8)
Hematopoietic cell lineage (11)	Hepatitis C (13)	Endocytosis (8)
Complement and coagulation cascades (11)	Malaria (12)	Protein processing in endoplasmic reticulum (8)
Natural killer cell-mediated cytotoxicity (10)	Amoebiasis (11)	Nucleotide excision repair (4)
Chemokine signaling pathway (9)	Microbial metabolism in diverse environments (10)	Spliceosome (4)
Phagosome (9)	Leishmaniasis (9)	DNA replication (3)
Lysosome (8)	Viral myocarditis (6)	Homologous recombination (3)
T cell receptor signaling pathway (8)	Staphylococcus aureus infection (6)	RNA transport (2)
Toll-like receptor signaling pathway (8)	Bacterial invasion of epithelial cells (4)	Mismatch repair (2)
NOD-like receptor signaling pathway—(7)	Pathogenic Escherichia coli infection (3)	Base excision repair (2)
Systemic lupus erythematosus (7)	Epithelial cell signaling in Helicobacter pylori infection (2)	*Apoptosis and oxidative stress*
B cell receptor signaling pathway (6)	Shigellosis (2)	Apoptosis (10)
Graft-versus-host disease (6)	*Metabolism*	Drug metabolism cytochrome P450 (6)
Cytosolic DNA-sensing pathway (5)	Oxidative phosphorylation (14)	Glutathione metabolism (5)
Antigen processing and presentation (5)	Arginine and proline metabolism (7)	Metabolism of xenobiotics by cytochrome P450 (5)
Intestinal immune network for IgA production (5)	Glycolysis/gluconeogenesis (5)	Peroxisome (3)
Type I diabetes mellitus (5)	Valine, leucine, and isoleucine degradation (4)	Drug metabolism—other enzymes (2)
Salivary secretion (5)	One carbon pool by folate (3)	*Signalling pathways*
Adipocytokine signaling pathway (5)	Terpenoid backbone biosynthesis (3)	MAPK signalling (35)
Fc epsilon RI signaling pathway (4)	Pyruvate metabolism (3)	Calcium signaling pathway (13)
Allograft rejection (4)	Citrate cycle (TCA cycle) (3)	PPAR signaling pathway (12)
TGF-beta signaling pathway (4)	Glycine, serine, and threonine metabolism (3)	Neurotrophin signaling pathway (12)
Autoimmune thyroid disease (4)	Protein digestion and absorption (3)	Wnt signaling pathway (10)
RIG-I-like receptor signaling pathway (4)	Tyrosine metabolism (3)	Insulin signaling pathway (9)
Jak-STAT signaling pathway (4)	Steroid hormone biosynthesis (3)	VEGF signaling pathway (7)
Fc gamma R-mediated phagocytosis (3)	Steroid biosynthesis (3)	Vascular smooth muscle contraction (6)
Leukocyte transendothelial migration (3)	Glycerolipid metabolism (3)	Notch signaling pathway (5)
Asthma (3)	Porphyrin and chlorophyll metabolism (3)	ABC transporters (5)
Primary immunodeficiency (2)	Histidine metabolism (2)	Renin-angiotensin system (4)
	Sphingolipid metabolism (2)	Cardiac muscle contraction (4)
	Cysteine and methionine metabolism (2)	mTOR signaling pathway (3)
	Purine metabolism (2)	ErbB signaling pathway (3)
	Tryptophan metabolism (2)	Aldosterone-regulated sodium reabsorption (3)
	Lysine degradation (2)	Progesterone-mediated oocyte maturation (3)
	Primary bile acid biosynthesis (2)	GnRH signaling pathway (2)
		Hedgehog signaling pathway (2)

**Table 2:** The relationships of Alzheimer's disease susceptibility genes with vitamin A. NF = none found.

Gene	Name	Relationships with vitamin A
*Transport and entry*		
ALB	Albumin	Together with retinol binding protein forms the retinol transporter [39]
APOE	Apolipoprotein E	Expression regulated by LXR/RXR dimers [40] Involved in retinyl palmitate transport [41]
TTR	Transthyretin (prealbumin, amyloidosis type I)	Carrier protein for the retinol binding protein [42]
APOA1	Apolipoprotein A-I	RORA target [43]: associates with transthyretin in plasma [44]
HSPG2	Perlecan: (heparan sulfate proteoglycan 2)	Binds to transthyretin [45]
A2M	Alpha-2-macroglobulin	Synthesis decreased in vitamin-A- deficient rats [46]
ABCA1	ATP-binding cassette, subfamily A (ABC1), member 1	22R-hydroxycholesterol and 9-cis-retinoic acid induce ABCA1 expression and cholesterol efflux in brain cells and decrease Beta-amyloid secretion [47]. Involved in retinol and alpha- and gamma-tocopherol transport [48, 49]
CLU	Clusterin (APOJ) LRP2 ligand	The clusterin promoter contains a RARE sequence: Expression is suppressed by all-trans-retinoic acid [50]. Vitamin A deficiency increases clusterin expression in sertoli cells [51]
LRP2	Low density lipoprotein-related protein 2 (clusterin receptor)	Mediates the endocytosis of retinol via binding to retinol binding proteins and transthyretin [52, 53]
LRPAP1	Low density lipoprotein receptor-related protein associated protein 1	Regulates the uptake of retinol by LRP2 [54]

*Metabolism*		
ALDH2	Aldehyde dehydrogenase 2 family (mitochondrial)	Exhibits low NAD(+)-dependent retinaldehyde activity [55]: regulated by RARB [56]
CYP46A1	Cytochrome P450, family 46, subfamily A, polypeptide 1	Synthesises 24-S hydroxycholesterol, a ligand for RARA and RARG [57]
GSTM1	Glutathione S-transferase M1	Weakly catalyses the enzymic isomerization of 13-cis-retinoic acid to all-trans-retinoic acid [58]
GSTP1	Glutathione S-transferase pi	Catalyses the enzymic isomerization of 13-cis-retinoic acid to all-trans-retinoic acid [58]
LIPA	Lipase A, lysosomal acid, cholesterol esterase (Wolman disease)	Metabolises carotenoid mono- and diesters providing a source of free carotenoids in the gut [59]
LPL	Lipoprotein lipase	Metabolises retinyl esters [60]. RORA target [61]
LRAT	Lecithin retinol acyltransferase (phosphatidylcholine—retinol O-acyltransferase)	Lecithin retinol acyltransferase (phosphatidylcholine—retinol O-acyltransferase)
MEF2A	Myocyte enhancer factor 2A	Regulates beta-carotene 15,15′-monooxygenase 1 which cleaves beta-carotene to all-trans retinal and is the key enzyme in the intestinal metabolism of carotenes to vitamin A [62]

*Receptors, coreceptors and receptor binding partners*		
CHD4	Chromodomain helicase DNA binding protein 4	Binds to RORG [63]
ESR1	Estrogen receptor 1	Dimerises with RAR and RXRA [52]
KLF5	Kruppel-like factor 5 (intestinal)	Binds to RARA [64]
NPAS2	Neuronal PAS domain protein 2	RAR alpha and RXR alpha bind to CLOCK and NPAS2 [65]. RORA target [66]
NR1H2	Nuclear receptor subfamily 1, group H, member 2: liver X receptor beta	LXRs form obligate heterodimers with retinoid X receptors RARA, RXRA, RXRB, RXRG (Entrez gene)
PARP1	Poly (ADP-ribose) polymerase family, member 1	Interacts with RARB [67]
PIN1	Protein (peptidylprolyl cis/trans isomerase) NIMA-interacting 1	RARalpha directly interacts with Pin1. Overexpression of Pin1 inhibits ligand-dependent activation of RARalpha [68]
POU2F1	POU class 2 homeobox 1	Binds to RXR [69]
PPARA	Peroxisome proliferator-activated receptor alpha	Dimerises with RXRA and RXRG receptors [70]
PPARG	Peroxisome proliferator-activated receptor gamma	Dimerises with RXRA receptors [71]
RXRA	Retinoid X receptor, alpha	Retinoic acid receptor
THRA	Thyroid hormone receptor, alpha (erythroblastic leukemia viral (v-erb-a) oncogene homolog, avian)	Dimerises with RXRA [72]
UBQLN1	Ubiquilin 1	Binds to retinoic acid receptor alpha [73]
VDR	Vitamin D (1,25-dihydroxyvitamin D3) receptor	Heterodimerises with RXR and RARG [74]

*APP and tau processing*		
APP	Amyloid beta (A4) precursor protein (peptidase nexin-II, Alzheimer disease)	A gamma 57 gamma secretase cleavage product suppresses retinoid signalling [75]
BACE1	Beta-site APP-cleaving enzyme 1	Regulated by all-trans-retinoic acid [76]
NCSTN	Nicastrin	Blocks the effects of retinoic acid on neurogenesis [77]
PSEN1	Presenilin 1 (Alzheimer disease 3)	Regulated by and regulates the effects of retinoic acid on neuronal differentiation [78, 79]
PSEN2	Presenilin 2 (Alzheimer disease 4)	Activated by all-trans-retinoic acid in osteoblasts [80]
CDK5	Cyclin-dependent kinase 5	Activated by retinoic acid [81]
GSK3B	Glycogen synthase kinase 3 beta	SH-SY5Y cells differentiate to neuron-like cells when treated with Retinoic acid/BDNF leading to increases in tau and tau phosphorylation, mediated primarily by GSK3B [82]: GSK3B inhibitors inhibit RARbeta-induced adipogenesis in mouse embryonic stem cells [83].
MAPT	Microtubule-associated protein tau	Phosphorylation of tau at the 12E8 (Ser-262/Ser-356) epitope decreased in retinoic acid treated cells: increased at Ser-195/Ser-198/Ser-199/Ser-202) and (Ser-396/Ser-404) [84]

*Viral and bacterial defence RIG-1, PKR, NOD and Toll receptor signalling*		
CARD8	Caspase recruitment domain family, member 8	NF
CD14	CD14 molecule	Expression regulated by retinoids [85]: binds to H. Pylori lipopolysaccharide [86].
CD86	CD86 molecule	Expression modulated by and the viral DNA minic polyriboinosinic:polyribocytidylic acid [87]
CST3	Cystatin C	NF antimicrobial peptide [88]
DEFB122	Defensin, beta 122: Antimicrobial peptide	NF
EIF2AK2	Eukaryotic translation initiation factor 2-alpha kinase 2: (PKR activated by viral DNA)	Upregulated by retinoic acid in HL-60 leukemia cells [89]
GBP2	Guanylate binding protein 2, interferon inducible	NF
MEFV	Mediterranean fever	NF
MPO	Myeloperoxidase	Antimicrobial peptide [88], expression regulated by RXR/PPAR gamma heterodimer [90]
PIN1	peptidylprolyl cis/trans isomerase, NIMA-interacting 1	Binds to and negatively regulates IRF3 [91]
TF	Transferrin	Antimicrobial peptide [88]. Vitamin A deficiency is associated with modified iron homeostasis that can be reversed by retinoid supplementation, TF contains a peroxisome proliferator-activated receptor-retinoic acid X receptor heterodimer binding site [92]
TRAF2	TNF receptor-associated factor 2	Infection with RNA viruses activates the cytoplasmic retinoic acid-inducible gene-I (RIG-I) pathway which activates transcription factor IRF-3 which in turn induces many antiviral genes. It also induces apoptosis via TRAF2 [25]
TLR4	Toll-like receptor 4	Expression suppressed by retinoic acid [93]
PVRL2	Poliovirus receptor-related 2 (herpesvirus entry mediator B)	NF: herpes simplex receptor
ZBP1	Z-DNA binding protein 1: DNA-dependent activator of interferon regulatory factors	NF

*Cholesterol lipoprotein networks and lipid rafts*		
ABCA1		Retinol transporter (see above)
ABCG1	ATP-binding cassette, subfamily G (WHITE), member 1	Expression regulated by 9-cis retinoic acid and 22-hydroxycholesterol [94]
APOA5	Apolipoprotein A-V	Regulated by RORA [95]. APOA5 polymorphisms modify lipoprotein bound retinyl palmitate concentrations [96]
APOC2	Apolipoprotein C-II	Expression regulated by 9-cis-retinoic acid [97].
APOC3	Apolipoprotein C-III	RORA target [43]
APOC4	Apolipoprotein C-IV	Expression regulated by RXR ligands [98]
APOD	Apolipoprotein D	Expression regulated by RARA [99]
CETP	Cholesteryl ester transfer protein, plasma	Expression induced by 9-cis retinoic acid (RXR agonist) [100]
FDPS	Farnesyl diphosphate synthase (farnesyl pyrophosphate synthetase, dimethylallyltranstransferase, geranyltranstransferase)	Activated by the LXR/retinoid X receptor dimer [101]
LPA	Lipoprotein, Lp(a)	Isotretinoin reduces LPA serum levels [102]
LDLR	Low-density lipoprotein receptor (familial hypercholesterolemia)	NF
LRP1	Low-density lipoprotein-related protein 1 (alpha-2-macroglobulin receptor)	NF
LRP2		See above (retinol receptor)
LRP6	Low-density lipoprotein receptor-related protein 6	Expression induced by retinoic acid [103]
LRP8	Low-density lipoprotein receptor-related protein 8, apolipoprotein e receptor	NF
NPC1	Niemann-Pick disease, type C1	NF
NPC2	Niemann-Pick disease, type C2	NF
OLR1	Oxidized low-density lipoprotein (lectin-like) receptor 1	NF
RFTN1	Raftlin, lipid raft linker 1	NF
SOAT1	sterol O-acyltransferase 1: cholesterol acyltransferase	NF
SREBF1	Sterol regulatory element binding transcription factor 1	Liver X receptor/RXR target [104]
VLDLR	Very-low-density lipoprotein receptor	All-trans retinoic acid increases expression in adenocarcinoma cells [105]

*Chemokines and cytokines and inflammation*		
AGER	Advanced glycosylation end product-specific receptor	Expression upregulated by retinol and vitamin A [106, 107]
ALOX5	Arachidonate 5-lipoxygenase	RORA target [108]
CCL2	Chemokine (C-C motif) ligand 2	All-trans-retinoic acid suppresses bacterial lipopolysaccharide-induced expression and release in astrocytes [109]
CCL3	Chemokine (C-C motif) ligand 3	See CCL2 above
CCR2	Chemokine (C-C motif) receptor 2	Expression regulated by 9-cis-Retinoic acid [110]
IL10	Interleukin 10	All trans-retinoic acid increases IL10 production in monocytes and macrophages [111]
IL18	Interleukin 18 (interferon-gamma-inducing factor)	Differentiation of SH-SY5Y neuroblastoma cells by all-trans retinoic acid activates IL18 [112].
IL1A	Interleukin 1, alpha	Retinoic acid decreases expression in thymic epithelial cells [113]
IL1B	Interleukin 1, beta	Intraperitoneal retinoic acid reduces IL-1*β*, IL-6 and TNF*α* mRNA levels in the spinal cord after injury [114]. All-trans-retinoic acid increases IL1B expression in human aortic smooth muscle cells [115]
IL33	Interleukin 33	NF
IL6	Interleukin 6 (interferon, beta 2)	Retinoic acid increases expression in thymic epithelial cells [113].
IL8	Interleukin 8	Retinoid administration decreases polymorphonuclear neutrophilic leukocyte accumulation in mammary alveoli activated by lipopolysaccharide, and decreases IL-8 serum levels [116]
IL1RN	interleukin 1 receptor antagonist	Retinoic acid enhances IL-1 beta and inhibited IL-1ra production in 4beta phorbol 12beta-myristate-13alpha acetate - and lipopolysaccharide-activated human alveolar macrophages [117].
PTGS2	Prostaglandin-endoperoxide synthase 2 (prostaglandin G/H synthase and cyclooxygenase)	Suppressed by RARB [118]
TGFB1	Transforming growth factor, beta 1	Repressed by RXRA.PPARG dimers [119]
TNF	Tumor necrosis factor (TNF superfamily, member 2)	LPS from bacterial pathogens activates Retinoic inducible gene RIG-I which plays a key role in the expression of TNF-alpha in macrophages in response to LPS stimulation [120]
FAS	Fas (TNF receptor superfamily, member 6)	Retinoic acid increases the expression of FAS in adipocytes: all-trans retinoid acid reduces FAS expression in HELA cells

*Complement and immune system*		
C4A	Complement component 4A (Rodgers blood group)	Complement C4 levels correlate with those of retinol in plasma [121]
C4B	Complement component 4B (Chido blood group)	See above
CFH	Complement factor H	Expression controlled by RAR beta [122]
CLU	Clusterin	See above
CR1	Complement component (3b/4b) receptor 1 (Knops blood group)	NF
CRP	C-reactive protein, pentraxin related	Serum CRP levels negatively correlate with vitamin A levels [123]
		
CD33	CD33 molecule	NF
CD36	CD36 molecule (thrombospondin receptor)	RORA target [61]
HLA-A	Major histocompatibility complex, class I, A	Upregulated by differentiation of teratoma cells into neuronal cells by retinoic acid [124]
HLA-A2	Major histocompatibility complex, class I, A2	Upregulated by interferon alpha-2b and retinoic acid combined treatment in cervical cancer cells [125]
MICA	MHC class I polypeptide-related sequence A	Expression upregulated by retinoic acid in hepatic carcinoma cells [126]

*Oestrogen and androgen related*		
AR	Androgen receptor (dihydrotestosterone receptor; testicular feminization; spinal and bulbar muscular atrophy; Kennedy disease)	
ESR1		See above
ESR2	Estrogen receptor 2 (ER beta)	9-cis retinoic acid stimulates expression in breast cancer cells [127]
CYP19A1	Cytochrome P450, family 19, subfamily A, polypeptide 1: aromatase: estrogen synthase	Activated by RORA [128]
HSD11B1	Hydroxysteroid (11-beta) dehydrogenase 1	NF

*Growth factor networks*		
BDNF	Brain-derived neurotrophic factor	Expression regulated by RARalpha/beta and vitamin A [129, 130], but all-trans retinoic acid reduces BDNF and TrkB gene expression in SH-SY5Y cells [131]
CSK	C-src tyrosine kinase	CSK negatively regulates RAR functions in relation to neurite differentiation [132]
FGF1	Fibroblast growth factor 1 (acidic)	Protects fibroblasts from apoptosis induced by retinoid CD437 [133]
GAB2	GRB2-associated binding protein 2	Gab2 silencing results in hypersensitivity to retinoic acid -induced apoptosis in neuronal cells [134]
IGF1	Insulin-like growth factor 1 (somatomedin C)	Pulmonary expression reduced in RORalpha knockout mice [135]
NTRK1	Neurotrophic tyrosine kinase, receptor, type 1	Retinoic acid restores adult hippocampal neurogenesis and reverses spatial memory deficit in vitamin-A-deprived rats, partly by upregulating NTRK1 (TrkA) [136]
NTRK2	Neurotrophic tyrosine kinase, receptor, type 2	All-trans retinoic acid reduces BDNF and TrkB gene expression in SH-SY5Y cells [131]
VEGFA	vascular endothelial growth factor A	Expression regulated by retinoid acid [137]
Other signalling		
DKK1	Dickkopf homolog 1 (Xenopus laevis)	Expression regulated by retinoic acid in stem cells [103]
DPYSL2	Dihydropyrimidinase-like 2	Upregulated in cortex and hippocampus by Vitamin A depletion [138]

*Homocysteine and methionine metabolism*		
BLMH	Bleomycin hydrolase	Hydrolyses homocysteine thiolactone [139]
CBS	Cystathionine-beta-synthase	Converts homocysteine to cystathionine suppressed by all-trans-retinoic acid [140]
MSRA	methionine sulfoxide reductase A	Regulated by retinoic acid via two promoters including RARA [141]
MTHFD1L	Methylenetetrahydrofolate dehydrogenase (NADP+ dependent) 1-like	NF
MTHFR	5,10-methylenetetrahydrofolate reductase (NADPH)	Methylenetetrahydrofolate reductase activity is suppressed in retinol-fed rats [142]
MTR	5-methyltetrahydrofolate-homocysteine methyltransferase	In rats, a retinol-rich diet enhances the folate-dependent oxidation to CO2 of formate and histidine. The activity of hepatic methylenetetrahydrofolate reductase, which regulates liver folate metabolism, is suppressed, leading to decreased 5-methyltetrahydrofolate synthesis [142]
MTRR	5-methyltetrahydrofolate-homocysteine methyltransferase reductase	NF
PON1	Paraoxonase 1	Hydrolyses homocysteine thiolactone [143]: vitamin A deficiency reduced serum PON1 activity in rats [144].

*Oxidative stress, Iron and mitochondria*		
COX1	Mitochondrially encoded cytochrome c oxidase I	9-cis retinoic acid treatment increases mitochondrial DNA transcription, including ND1, ND6, and COX1 [145]
COX2	Mitochondrially encoded cytochrome c oxidase II	Expression increased by all-trans retinoic acid [146]
GAPDH	Glyceraldehyde-3-phosphate dehydrogenase	Retinoic acid target [147]
GSTM3	Glutathione S-transferase M3 (brain)	Contains a retinoid X receptor-binding site [148]
HBG2	Hemoglobin, gamma G	Vitamin A increases haemoglobin concentrations in children [149]
HFE	hemochromatosis	Neuroblastoma cells carrying the C282Y HFE variant do not differentiate when exposed to retinoic acid [150]
HMOX1	Heme oxygenase (decycling) 1	The increase in the expression of heme oxygenase-1 and the growth arrest and DNA damage-inducible transcription factor 153 caused by reactive oxygen species is blocked by aRARalpha-specific antagonist AGN194301 in retinal epithelial cells [151]
ND1	NADH dehydrogenase subunit 1	9-cis retinoic acid treatment increases mitochondrial DNA transcription, including ND1, ND6, and COX1 [145]
ND4	NADH dehydrogenase subunit 4	Upregulated by all-trans retinoic acid in neutrophils [152]
ND6	NADH dehydrogenase subunit 6	9-cis retinoic acid treatment increases mitochondrial DNA transcription, including ND1, ND6, and COX1 [145]
NFE2L2	Nuclear factor (erythroid-derived 2)-like 2	Inhibited by retinoic acid via RARalpha resulting in lack of expression of Nrf2 target genes [153] in mammary cells, but retinoic acid and 12-O-tetradecanoylphorbol acetate are also able to induce Nrf2 and its target gene NAD(P)H quinone oxidoreductase 1 in the SH-SY5Y neuroblastoma cell line [154].
NOS1	Nitric oxide synthase 1 (neuronal)	Expression regulated by retinoic acid [84]
NOS2	Nitric oxide synthase 2, inducible	Ditto
NOS3	Nitric oxide synthase 3 (endothelial cell)	Ditto
NQO1	NAD(P)H dehydrogenase, quinone 1	Retinoic acid (RA) and 12-O-tetradecanoylphorbol acetats are able to induce Nrf2 and its target gene NAD(P)H quinone oxidoreductase 1 in the SH-SY5Y neuroblastomacell line [154]
SOD2	Superoxide dismutase 2, mitochondrial	All-trans-retinoic acid induces manganese superoxide dismutase in a human neuroblastoma cell line [155]
PCK1	Phosphoenolpyruvate carboxykinase 1 (soluble)	Three RXR-binding elements (retinoic acid response element (RARE)1/PCK1, RARE2, and RARE3/PCK2) are located in the promoter of Pck1 [156]
PON2	Paraoxonase 2	NF
PON3	Paraoxonase 3	NF
TFAM	Transcription factor A, mitochondrial	Levels are increased by vitamin A [157]

*Heat shock*		
DNAJC28	DnaJ (Hsp40) homolog, subfamily C, member 28	NF
HSPA1B	Heat shock 70 kDa protein 1B	NF
HSPA5	Heat shock 70 kDa protein 5 (glucose-regulated protein, 78 kDa)	Endoplasmic reticulum stress is increased in hepatocarcinoma cells by all-trans retinoic acid, characterised by increased expression of HSPA5 (grp78), GADD153, and XBP1[158]

*Monoamine networks*		
ADRB1	Adrenergic, beta-1-, receptor	RARA target [159]
ADRB2	Adrenergic, beta-2-, receptor, surface	Expression regulated by all-trans retinoic acid [160]
COMT	Catechol-O-methyltransferase	Expression stimulated by all-trans retinoic acid [161]
PNMT	Phenylethanolamine N-methyltransferase	Retinoic acid differentiates embryonic carcinoma cells into neuronal cells, 70% of which stain for tyrosine hydroxylase, dopamine beta-hydroxylase, and phenylethanolamine N-methyltransferase [162].

*Cholinergic*		
CHAT	Choline acetyltransferase	Expression controlled by retinoic acid [163]
CHRNA3	Cholinergic receptor, nicotinic, alpha 3	Expression increased by retinoid acid [164]
CHRNA4	Cholinergic receptor, nicotinic, alpha 4	Expression decreased by retinoic acid [164]
CHRNB2	Cholinergic receptor, nicotinic, beta 2 (neuronal)	Expression increased by retinoid acid [164]

*Neuropeptide*		
GRN	Granulin	All-trans retinoic acid increases expression in myeloid cells [165]

*Adhesion and proteoglycans*		
ACAN	Aggrecan	Expression modulated by 13-cis retinoic acid in fibroblasts [166]
ICAM1	Intercellular adhesion molecule 1 (CD54), human rhinovirus receptor	All-trans retinoic acid downregulates ICAM1 expression in bone marrow stromal cells [167]

*Structural, dynamins, and kinesins*		
COL11A1	Collagen, type XI, alpha 1	Expression controlled by all-trans-retinoic acid [168]
DSC1	Desmocollin 1	Retinoic acid decreases expression in oral keratinocytes [169]
LMNA	Lamin A/C (nuclear)	Promoter contains a retinoic acid-responsive element (L-RARE) [170]

*Ubiquitin*		
UBD	Ubiquitin D	All-trans retinoic acid activated the ubiquitin/proteasome pathway in human acute myeloid leukemia cell lines [171]
UBE2I	Ubiquitin-conjugating enzyme E2I (UBC9 homolog, yeast)	See above

*DNA repair*		
XRCC1	X-ray repair complementing defective repair in Chinese hamster cells 1	N-[4-hydroxyphenyl] retinamide induces apoptosis in bladder cancer cell line and downregulates XRCC1 [172]

*Cell cycle and tumour*		
CDC2	Cell division cycle 2, G1 to S, and G2 to M	Activated by retinoic acid [173]

*Miscellaneous metabolism*		
ACAD8	Acyl-coenzyme A dehydrogenase family, member 8	Catalyzes the dehydrogenation of acyl-CoA derivatives in the metabolism of fatty acids or branch chained amino acids. The encoded protein is a mitochondrial enzyme that functions in catabolism of the branched-chain amino acid valine.
ALDH18A1	Aldehyde dehydrogenase 18 family, member A1	NF the encoded protein catalyzes the reduction of glutamate to delta1-pyrroline-5-carboxylate, a critical step in the de novo biosynthesis of proline, ornithine and arginine.
ARSA	Arylsulfatase A: hydrolyzes cerebroside sulfate to cerebroside and sulphate	Increased urinary excretion of both arylsulfatases A and B is increased in cases of severe vitamin A deficiency coupled with malnutrition [174]
ELAVL4	ELAV (embryonic lethal, abnormal vision, drosophila)-like 4 (Hu antigen D)	Inhibition reduces retinoic acid-induced neuronal differentiation of mouse embryonal carcinoma P19 cells [175]
SGPL1	Sphingosine-1-phosphate lyase 1	Treatment of F9 embryonal carcinoma cells with retinoic acid induces differentiation to primitive endoderm (PrE). This effect is attenuated by SGPL1 knockout [176].

*Miscellaneous*		
CELF2	CUGBP, Elav-like family member 2	Splicing regulated by retinoic acid [177]
CUBN	Cubilin (intrinsic factor-cobalamin receptor)	Expression regulated by all-trans-retinoic acid [178]
F13A1	Coagulation factor XIII, A1 polypeptide	Vitamin A reduces factor XIII levels in rats fed an atherogenic diet [179].
HHEX	Hematopoietically expressed homeobox	All-trans retinoic acid enhances expression in normal and tumorous mammary tissue [180]
NEDD9	Neural precursor cell expressed, developmentally downregulated 9	Downstream target of all-trans retinoic acid and its receptors in the human SH-SY5Y neuroblastoma cell line [181]
RELN	Reelin	Retinoic acid-induced differentiation of NT2 cells to hNT neurons increases reelin expression [182].
RNR1	RNA, ribosomal 1	NF
RPS3A	Ribosomal protein S3A	Downregulated by retinoid-induced differentiation of HL-60 cells [183]
RUNX1	Runt-related transcription factor 1 (acute myeloid leukemia 1; aml1 oncogene)	RUNX1 knockdown inhibits retinoid-induced differentiation of HL-60 myeloid leukaemia cells [184].
SEPT3	Septin 3	Expressed in SH-SY5Y, after retinoic acid-induced differentiation [185]
SNCA	Synuclein, alpha (non-A4 component of amyloid precursor)	Vitamin A, beta-carotene and coenzyme Q10 inhibit the formation of synuclein fibrils [186]
SYN3	Synapsin III	Synapsins including SYN3 are upregulated by retinoic acid-induced differentiation of NTera-2cl.D1 cells [187]
TARDBP	TAR DNA binding protein	NF
VCP	Valosin-containing protein	Retinoic acid receptor responder (RARRES1) regulates VCP expression in human prostatic epithelial cells [188]

**Table 3:** Viral, bacterial, and fungal protein homology with beta-amyloid; beta-amyloid segments were compared with Borrelia Burgdorferi, C. Neoformans, C. Pneumoniae, H. Pylori, P. Gingivalis, and herpes viruses (HSV1, HHV6, and cytomegalovirus) proteomes by BLAST analysis. The B cell and T cell antigenicity indices are shown, and those above the server set threshold of 0.35 (B cell epitope) or 0.5 (T cell epitope) are highlighted in bold. The first column shows the amino acid sequence of beta-amyloid_1-42_ and the alignments with pathogen proteins are shown. Spaces represent nonidentical amino acids and + signs amino acids with similar physicochemical properties. Only highly antigenic regions of pentapeptides or more were processed. The VGGVV sequence, antibodies to which label beta-amyloid in brain tissue, despite relatively low antigenicity, has already been reported to be identical to proteins expressed by 69 viruses including HSV-1, HSV-2, and HHV6 [230].

	B cell	T cell	Alignments
D	**0.41**	0.04	+AE HDSG+ C. Neoformans DAE F H+SG EV Borrelia burgdorferi DAEF H. Pylori DA FRH H. Pylori +AE RH HSV-1 D FR DS HHV-6 +AEFR P. Gingivalis DA EFRHD and +AEFR +SG C. Pneumoniae
A	**0.35**	**0.78**	AEFR D GY+V C. Neoformans AEF D S YE H. pylori AE+RH+ H. pylori AEF H+ Borrelia burgdorferi AEFR HD Cytomegalovirus AE R SG HSV-1 AE+ HD HHV-6 A+F H+S and AEFR P. Gingivalis AEF DSG C. Pneumoniae
E	**0.62**	0.02	EFRHD H. Pylori EF DSG HHV-6 EFR DS Borrelia Burgdorferi EF R DS YE C. Neoformans E R DSGY V P. Gingivalis EF SGYEV C. Pneumoniae
F	**0.73**	**0.93**	FRHDS C. Neoformans +RH SGY++ Borrelia burgdorferi F HD EV H. Pylori F H+SG H. Pylori FR SGY Cytomegalovirus +RHDS P. Gingivalis F H+SGY C. Pneumonia
R	**0.85**	0.05	R D GYEV C. Neoformans R SGYE H. pylori RHDS Y V H. pylori RH+ GY Borrelia burgdorferi RHDSG Cytomegalovirus RHD YE cytomegalovirus RH SG HSV-1 RHDS HHV-6 R+DS Y+ P. Gingivalis
H	**0.57**	0.14	HDSGY C. Neoformans HD G EV H. pylori +DSGY H. pylori H+SG Y+V Borrelia burgdorferi H+SG HSV-1 HDSG P. Gingivalis ++SGY+V C. Pneumoniae
D	**0.69**	0.03	DSGY+V C. Neoformans +SG+EV H. pylori DSGY HSV-1 DSG+EV P. Gingivalis DSGY V C. Pneumoniae
S	**0.38**	0.02	SGYEV P. Gingivalis SGYE H. pylori SGY++ C. Neoformans SG+EV C. Pneumoniae
G	**0.63**	0.04	GYEVH H. pylori GYE V KL+ Borrelia Burgdorferi GYE LV and GY++ + LV C. Neoformans GYEV P. Gingivalis GYEV and GY HH C. Pneumoniae
Y	**0.56**	**0.96**	YE HH and YE+ HQ and Y++H Q and YE HHQ H. pylori YEVH Cytomegalovirus YE+ KL Borrelia Burgdorferi YE + QK FC. Neoformans Y++H H+K P. Gingivalis Y+V +Q LV C. Pneumoniae
E	**0.58**	0.02	EV +QK H. pylori EV HQ L Cytomegalovirus EV +KL Borrelia Burgdorferi EV Q LV C. Neoformans EV KLV P. Gingivalis EV QKLV C. Pneumoniae
V	0.35	**0.90**	.+H QK H. pylori V HQ LV Cytomegalovirus VH QK+V HHV-6 VH KL Borrelia Burgdorferi +HH LV C. Neoformans VH + LV P. Gingivalis V HQKL C. Pneumoniae
H	−0.17	0.10	HHQK H. pylori and C. Pneumoniae HH KL Cytomegalovirus HH KL P. Gingivalis
H	−0.66	0.11	HQKL+ Borreli Burgdorferi HQKL C. Pneumoniae and HSV-1 +QKLV P. Gingivalis
Q	−1.03	0.03	QKLV C. Neoformans and P. Gingivalisand C. Pneumoniae
K	−1.47	**0.93**	KLVFF H. pylori: Cryptococcus neoformans Borrelia Burgdorferi Chlamydophila pneumoniae KLVF Human herpesvirus 1
L	−1.34	**0.95**	LVFF Human herpesvirus 5: Human herpesvirus 6
V	−1.20	**0.68**	
F	−0.93	**0.69**	
F	−0.98	**0.74**	
A	−0.82	0.05	
E	−0.31	0.05	
D	0.23	0.11	
**V**	**0.81**	**0.88**	VGSNK Borrelia burgdorferi Cryptococcus neoformans Porphyromonas gingivalis +GSNK Cytomegalovirus VGSN Helicobacter pylori Chlamydophila pneumoniae
**G**	**1.24**	0.03	GSNK Helicobacter Pylori Chlamydophila pneumoniae
**S**	**1.22**	0.03	
**N**	**0.90**	0.03	
**K**	**0.36**	0.30	
G	0.30	0.10	
A	−0.24	0.05	
I	−0.58	0.07	
I	−1.00	0.13	
G	−1.14	0.03	
L	−1.19	**0.72**	
M	−1.23	0.13	
V	−1.16	0.23	VGGVV 69 viruses/phages
G	−0.97	0.03	
G	−1.02	0.03	
V	−0.63	0.23	
V	−0.45	0.33	
I	−0.80	**0.95**	
A	−1.06	**0.83**	
